# Information and communication technology literacy, knowledge and readiness for electronic medical record system adoption among health professionals in a tertiary hospital, Myanmar: A cross-sectional study

**DOI:** 10.1371/journal.pone.0253691

**Published:** 2021-07-01

**Authors:** Hlaing Min Oo, Ye Minn Htun, Tun Tun Win, Zaw Myo Han, Thein Zaw, Kyaw Myo Tun

**Affiliations:** 1 Outpatient Department, Defence Services Liver Hospital, Yangon, Myanmar; 2 Department of Prevention and Research Development of Hepatitis, AIDS and Other Viral Diseases, Health and Disease Control Unit, Nay Pyi Taw, Myanmar; 3 Department of Preventive and Social Medicine, Defence Services Medical Academy, Yangon, Myanmar; 4 Special Operation Medical Research Department, Defence Services Medical Research Centre, Nay Pyi Taw, Myanmar; Imam Abdulrahman Bin Faisal University, SAUDI ARABIA

## Abstract

Some developing countries are currently introducing and implementing an electronic medical record system (EMRs) for improvement in healthcare delivery services. Availability of information and communication technology (ICT), technical skillful staff, and strong resistance to change by the health professionals impacted the successful adoption of EMRs. This study aimed to assess the ICT literacy, knowledge, and readiness for EMRs adoption among health professionals in a tertiary hospital, Myanmar. A cross-sectional study was conducted among 118 health professionals involving in a tertiary hospital at Nay Pyi Taw, Myanmar from February to April 2020 using a stratified sampling method. The data were collected through face-to-face interviews using a pretested structured questionnaire after getting informed consent. Data were analyzed by using SPSS version 23.0. Chi-square test, Fisher’s exact test, and logistic regression analysis were performed to assess the associated factors of ICT literacy, knowledge, and overall readiness for EMRs adoption. The prevalence of high ICT literacy and knowledge on EMRs among health professionals were 20.3% and 24.6% respectively. The factors associated with ICT literacy were professional, education, duration of service, and reported English language skills. Duration of service was associated with knowledge on EMRs. The overall readiness was 54.2% (core readiness 59.3% and engagement readiness 61.9%), and postgraduate [Adjusted Odds Ratio (AOR): 7.32, 95% Confidence Interval (CI): 2.26–23.68] and knowledge on EMRs (AOR: 1.27, 95% CI: 1.13–1.43) were the factors associated with overall readiness for EMRs adoption. Expanding infrastructure and provision of ICT development training are crucial for the improvement of ICT literacy. EMRs training program enabling hands-on experience should be implemented for improvement of knowledge on EMRs. In general, the overall readiness for EMRs adoption was found to be moderate. Enhancing the establishment of comprehensive on-the-job training and contextualization of curriculum in EMRs training program are recommended to improve the health professionals’ readiness for EMRs adoption.

## Introduction

Information and communication technology (ICT) has great potential to improve the quality of health services in both developed and developing countries by enhancing the accessibility of health information and making efficient health services provision [1]. The electronic medical record system (EMRs) is being progressively introduced in healthcare settings of high-income countries. The adoption of EMRs has been steady growth over the past 15 years and a 46% global increase in the past five years. However, only 35% in the lower-middle and 15% of low-income countries have nationally adopted electronic record systems in health institutions [2].

The EMRs are in-house electronic versions of the traditional paper charts that display the patient information with the accessibility of evidence-based decision support tools used in decision-making by the clinicians [1, 3]. It is also a technology that allows a health professional’s practices to pursue the higher quality improvement of electronic health services than the paper-based health records [4]. The system was used for patient registration, medications and drug prescriptions, and recordkeeping in pharmaceutical tasks, laboratory results, and all healthcare information of the patients during each visit [1, 5]. Moreover, it could be applied in the data collection of public health disease surveillance and reporting [1].

The effective adoption of EMRs would provide health professionals through the use of comprehensive and timely patient information resulting in more accessible and better health care for a path towards universal health coverage [3]. The EMRs contributed many benefits including easy access to data, improved data collection, increased staff productivity, increased patients’ satisfaction with services, and improved communication [1, 6]. The system could also improve the healthcare services by increasing adherence to therapeutic guidelines and protocols, informing clinical decisions, and decreasing medication errors [7].

Therefore, the healthcare quality improvement could be made through the usage of the EMRs in the healthcare services [4]. The success of EMRs was demonstrated by improved quality of healthcare [1]. Establishing EMRs might provide an opportunity to define a core data set of the patients, to assist the local healthcare providers, and to improve the local health systems [8]. In developing countries, the Ministries of Health often require the healthcare facilities to collect and report a patient’s information from each visit, to track the healthcare costs, and to observe the main outcomes. There was progress of replacing paper-record with EMRs, only a few healthcare facilities have been successful [5, 9]. However, more than 50% of electronic systems were not successful in proper utilization [4, 10].

A better understanding of the usefulness of EMRs was an important factor for the preparation of EMRs [11]. The readiness assessment was the most important step prior to implementation and an essential requirement for the success of EMRs in terms of adoption rate or acceptance [12]. It could help healthcare facilities to identify barriers, to be successful EMR system adoption, and to measure the preparedness of the organization for available resources [13]. However, the resistance to change from the paper-based systems to electronic systems [14], lack of pre-implementation activities [10], lack of organizational readiness [15], poor readiness of healthcare professionals, unavailability of technology, limited funding, and lack of skilled technical staffs still existed as the problems for adoption [5, 12].

In Myanmar, the use of paper-based medical records is a common practice in hospitals and clinics [14, 16]. As a part of the overall health information management system, the Ministry of Health and Sports is implementing the District Health Information System to develop the electronic hospital reporting system and open medical record system for patient recording [17]. Therefore, the assessment of readiness in health professionals of a first initiated tertiary hospital is crucial for successful implementation. So far, there have been no attempts to investigate the predictors of ICT literacy and knowledge on EMRs in the health professionals, and very few studies have assessed the readiness for EMRs adoption. This study aimed to assess the information communication technology literacy, knowledge, and readiness for EMRs, and the associated factors among the health professionals.

## Materials and methods

### Study area and design

A cross-sectional study was conducted among health professionals in a tertiary hospital, No.2 1000 Bedded Hospital (Nay Pyi Taw) that situated in Nay Pyi Taw Union Territory, from February to April 2020. Nay Pyi Taw, the administrative capital of the Republic of the Union of Myanmar, is located 327 km from Yangon Region. Myanmar is one of the lower middle income countries [18].

### Study population

There were 436 health professionals (161 doctors and 275 nurses) in this hospital. The study population included doctors (including specialists and medical officers) and nurses (including senior or chief nurses, staff-nurses, and trained nurses). The doctors and nurses with less than 6 months of service duration, those who were on leave, those who were travelling, and those who were in training at the time of data collection, were excluded from the study.

### Sample size determination and sampling procedure

A total sample size of 118 participants was achieved using a single population proportion formula for quantitative studies [19], assuming 47.1% proportion of overall readiness for EMRs implementation in health care workers from a previous study in Myanmar [20], at a 9% margin of error and 95% confidence interval. The No.2 1000 Bedded Hospital (Nay Pyi Taw) was purposively selected as a tertiary hospital with the introduction of EMRs adoption. Stratified sampling method was then used to select the participants from each of the professional (doctors and nurses). According to the appointed doctor-nurse ratio, 1:2 at the targeted hospital, 39 doctors and 79 nurses were selected proportionately. A simple random sampling technique was used to select the participants in each professional.

### Data collection technique and procedure

A pretested structured questionnaire was administered to the health professionals, and it was prepared based on WHO EMRs readiness evaluation framework and additional literature related to readiness for EMRs [5, 6, 11]. The questionnaire consisted of 5 items for sociodemographic characteristics, reported English language skills, 12 items focused on ICT literacy, and 14 items about knowledge on EMRs. The score for English language skills of respondent were assigned “basic” as 1, “intermediate” as 2, and “advanced” as 3 for each skill and then the total score was range from 4 to 12. There were single response and multiple response items in both ICT literacy and knowledge on EMRs parts. For the scoring system of the “yes” or “no” or “don’t know” option, the correct responses were scored as one point, and incorrect responses or “don’t know” were scored as zero points. In multiple response items, one response was scored as one point. The readiness was assessed with 13 items for core readiness and 12 items for engagement readiness, by using the readiness assessment framework [21]. A four-point Likert scale was used for scoring the items of core and engagement readiness for EMRs adoption: strongly agree (4 points), agree (3 points), disagree (2 points), and strongly disagree (1 point).

The questionnaire was prepared in English and translated into the Burmese language, the local language. Before the data collection, a pilot study was conducted in 45 health professionals (15 doctors and 30 nurses) of No.1 1000 Bedded Hospital (Mingaladon) to test the internal consistency of all types of questions and their subscales assessed using Cronbach’s α. The homogeneity of the questionnaire was fair to strong with high Cronbach’s α ranging from 0.79 to 0.84. The ambiguous questions were revised or removed, and the questionnaire with local language was used to collect data after refining for improvement of validity. The questionnaire was, therefore, tolerable for its consistency in repeating what has been previously measured by the tools. The participants of the pilot study were not included in the final study.

### Operational definition

Health professionals were defined as medical doctors and nurses who were the main users of medical records in a health care setting, and they played a critical role in improving access and quality of health care for the population. The adoption of EMRs was a transformation of paper-based medical record systems to EMRs in a healthcare organization to improve the quality of healthcare. The education of health professionals was a designation of recent degrees for careers as graduate and postgraduate in the field of respective professionals.

Reported English language skills was a self-recognized level of English skills on reading, writing, listening, and speaking as a level of basic, intermediate, and advanced. The reported English language skills of the health professionals was classified as two categories, low and high, by using median cutoff value. ICT literacy was defined as the computer or technological knowledge in a capacity to obtain, communicate, process, and understand the basic health information and services in order to make appropriate health decisions [22]. The knowledge on EMRs was defined as knowledge about the electronic record of health-related information on a patient that could be created, collected, managed, and consulted by health professionals within a healthcare organization. The scores of ICT literacy and knowledge on EMRs were categorized as low (below 25^th^ percentile), medium (between 25^th^ and 75^th^ percentile), and high (above 75^th^ percentile).

Core readiness referred to the realization of needs for the services and along with an expressed dissatisfaction with existing service or circumstance [23–25]. Core readiness assessment result was determined by inefficient documentation of patient records, breached patient privacy, sharing of patient records, and healthcare providers’ dissatisfaction with completeness and accuracy [21]. The engagement readiness referred to the active willingness and participation of people in the idea of EMRs, the processing with the assessment of the risks, and the advantages and disadvantages of EMRs [24]. The engagement readiness assessment result was dependent on the concern about potential negative impacts, recognition of benefits, and willingness to accept EMRs [21]. Overall readiness was defined as the intersection of core readiness and engagement readiness. The health professionals with below the median score were categorized as not ready while those at or above the median score were considered as ready for adoption.

### Data quality control

The two interviewers participated in the data collection process. An one day intensive training was given to the interviewers on the objective of the study, data collection procedures, and tools. Daily supervision, completeness, and consistency of collected data were performed by the supervisors. Any confusion on the data collection procedure and responses were handled timely. Data back-up activities, like storing data at different places and putting data in different formats (hard and soft copies) were performed to prevent data loss.

### Data analysis

After cleaning and entering the data into an Excel 2016 spreadsheet, it was exported to the IBM SPSS Statistics for Windows, Version 23.0 (Armonk, NY: IBM Corp) for analysis. The normality of the continuous data was viewed by using the Kolmogorov-Smirnov test and the data distribution was non-normal. Categorical variables were presented as frequency (percentages) and analyzed by chi-square (χ^2^) test and Fisher’s exact test. Continuous variables were described as median (interquartile range, IQR) and compared using Kruskal-Wallis tests. The figure was generated by GraphPad Prism 8.0. To analyze the associated factors of overall readiness for EMRs adoption, binary logistic regression analysis was performed after checking the model fitness by the Hosmer and Lemeshow goodness of fit test. All independent variables in the bivariable analysis (sex, age, professional, education, duration of service, reported English language skills, ICT literacy, and knowledge on EMRs) were exported to a multivariable logistic regression model to control the potential effects of confounders. Crude odds ratio (COR) and adjusted odds ratio (AOR) with 95% confidence interval (CI) were calculated to determine the strength of association between independent variables and outcome variable, overall readiness on EMRs adoption. The potential differences in the effect of education for the readiness for EMRs adoption by knowledge on EMRs were assessed by likelihood ratio tests for interaction. For all analyses, the level of statistical significance was determined at p value < 0.05.

### Ethical consideration

Ethical approval was obtained from the Ethical Review Committee, Defence Services Medical Academy, Yangon Region, Myanmar, and permission was obtained from the administrative person of No.2 1000 Bedded Hospital (Nay Pyi Taw) for data collection. The study objectives, risks, and benefits were explained to all study participants before conducting the face-to-face interview. All participants provided the written informed consent including a right of withdrawing from this study without any restrictions. All data were anonymized to maintain the confidentiality of the participants.

## Results

In this study, 118 participants enrolled with a 100% response rate, using the structural questionnaires. Among the participants, almost, 92.4%, were male and 66.9% were nurses. The median (IQR) age of the health professionals was 30.00 (7.25, 28.00–35.25) years and 57.6% were 30 years and elders. Regarding the education of participants, 61.0% were graduates while 39.0% were postgraduates in their professionals. The median (IQR) duration of service was 10.00 (7.00, 7.00–14.00) years and 41.6% of the participants involved 6–10 years in service. Of all participants, 44.1% reported that they had a high level of English language skills and the median (IQR) score of reported English language skills was 4.00 (4.00, 4.00–8.00). In ICT literacy, the median (IQR) score was 34.50 (15.00, 24.00–39.00) and 20.3% of the participants had high ICT literacy. Concerning knowledge on EMRs, the median (IQR) score was 14.00 (5.25, 10.00–15.25) and 24.6% of the participants were in a high level of knowledge on EMRs (Table 1).

**Table 1:** Personal characteristics of health professionals.

Variables			n (%)	Median (IQR)
**Personal characteristics**				
	Sex			
		Male	109 (92.4)	
		Female	9 (7.6)	
	Age (year)			30.00 (7.25, 28.00–35.25)
		< 30	50 (42.4)	
		≥ 30	68 (57.6)	
	Professional			
		Nurse	79 (66.9)	
		Medical doctor	39 (33.1)	
	Education			
		Graduate	72 (61.0)	
		Postgraduate	46 (39.0)	
	Duration of service (year)			10.00 (7.00, 7.00–14.00)
		≤ 5	22 (18.6)	
		6–10	49 (41.6)	
		> 10	47 (39.8)	
	Reported English language skills			4.00 (4.00, 4.00–8.00)
		Low	66 (55.9)	
		High	52 (44.1)	
**ICT literacy**				
	Level of ICT literacy			34.50 (15.00, 24.00–39.00)
		Low	24 (20.3)	
		Medium	70 (59.4)	
		High	24 (20.3)	
**Knowledge on EMRs**				
	Level of knowledge			14.00 (5.25, 10.00–15.25)
		Low	22 (18.6)	
		Medium	67 (56.8)	
		High	29 (24.6)	

In this study, several factors were studied concerning the factors associated with ICT literacy and knowledge on EMR among health professionals. The factors associated with ICT literacy were shown in Table 2. A significantly higher ICT literacy score was seen in medical doctors compared to nurses (χ^2^ = 15.39, p value < 0.001), in postgraduates compared to the graduates (χ^2^ = 14.81, p value = 0.001), the health professionals with 6–10 years of service duration compared to other categories (Fisher’s exact = 9.89, p value = 0.039), and in those who had high reported English language skills compared to the health professionals with low English language skills (χ^2^ = 26.54, p value < 0.001).

**Table 2:** Factors associated with ICT literacy among health professionals.

Variables		Total	ICT literacy			χ^2^ / Fisher’s exact	p value
			Low	Medium	High		
			n (%)	n (%)	n (%)		
**Sex**							
	Female	9	1 (11.1)	6 (66.7)	2 (22.2)		
	Male	109	23 (21.1)	64 (58.7)	22 (20.2)	0.46	0.897 *
**Age**							
	< 30 years	50	7 (14.0)	32 (64.0)	11 (22.0)		
	≥ 30 years	68	17 (25.0)	38 (55.9)	13 (19.1)	2.15	0.341
**Professional**							
	Nurse	79	18 (22.8)	53 (67.1)	8 (10.1)		
	Medical doctor	39	6 (15.4)	17 (43.6)	16 (41.0)	15.39	< 0.001
**Education**							
	Graduate	72	21 (29.2)	43 (59.7)	8 (11.1)		
	Postgraduate	46	3 (6.5)	27 (58.7)	16 (34.8)	14.81	0.001
**Duration of service**							
	≤ 5 years	22	3 (13.6)	16 (72.7)	3 (13.6)		
	6–10 years	49	7 (14.3)	26 (53.1)	16 (32.7)		
	> 10 years	47	14 (29.8)	28 (59.6)	5 (10.6)	9.89	0.039 *
**Reported English language skills**							
	Low	66	20 (30.3)	43 (65.2)	3 (4.5)		
	High	52	4 (7.7)	27 (51.9)	21 (40.4)	26.54	< 0.001

^*^ p value by Fisher’s exact test, ICT: Information Communication and Technology

The factors associated with knowledge on EMRs were summarized in Table 3. The health professional with more than 10 years of service duration were found to have higher score of knowledge on EMRs compared to other categories of service duration and its association was statistically significant (Fisher’s exact = 14.09, p value = 0.006).

**Table 3:** Factors associated with knowledge on EMRs among health professionals.

Variables		Total	Knowledge on EMRs			χ^2^ / Fisher’s exact	p value
			Low	Medium	High		
			n (%)	n (%)	n (%)		
**Sex**							
	Female	9	0 (0.0)	6 (66.7)	3 (33.3)		
	Male	109	22 (20.2)	61 (56.0)	26 (23.9)	2.14	0.356 *
**Age**							
	< 30 years	50	9 (18.0)	33 (66.0)	8 (16.0)		
	≥ 30 years	68	13 (19.1)	34 (50.0)	21 (30.9)	3.92	0.141
**Professional**							
	Nurse	79	19 (24.1)	42 (53.2)	18 (22.8)		
	Medical doctor	39	3 (7.7)	25 (64.1)	11 (28.2)	4.61	0.100
**Education**							
	Graduate	72	17 (23.6)	37 (51.4)	18 (25.0)		
	Postgraduate	46	5 (10.9)	30 (65.2)	11 (23.9)	3.40	0.182
**Duration of service**							
	≤ 5 years	22	3 (13.6)	18 (81.8)	1 (4.5)		
	6–10 years	49	7 (14.3)	31 (63.3)	11 (22.4)		
	> 10 years	47	12 (25.5)	18 (38.3)	17 (36.2)	14.09	0.006 *
**Reported English language skills**							
	Low	66	16 (24.2)	36 (54.5)	14 (21.2)		
	High	52	6 (11.5)	31 (59.6)	15 (28.8)	3.34	0.188

^*^ p value by Fisher’s exact test, EMRs: Electronic Medical Record System

Of these participants, the median (IQR) score of core readiness was 38.00 (4.50, 35.75–40.25) and 59.3% of participants had core readiness. For the engagement readiness, the median (IQR) score was 34.00 (3.00, 32.00–35.00) and 61.9% of the health professionals had engagement readiness. In regards to overall readiness, the median (IQR) score was 72.00 (11.00, 66.00–77.00) and 54.2% of the health professionals were in overall readiness (Table 4).

**Table 4:** Readiness for EMRs adoption among health professionals.

Variables		n (%)	Median (IQR)
Core readiness			38.00 (4.50, 35.75–40.25)
	Not ready	48 (40.7)	
	Ready	70 (59.3)	
Engagement readiness			34.00 (3.00, 32.00–35.00)
	Not ready	45 (38.1)	
	Ready	73 (61.9)	
Overall readiness			72.00 (11.00, 66.00–77.00)
	Not ready	54 (45.8)	
	Ready	64 (54.2)	

**Fig 1:**
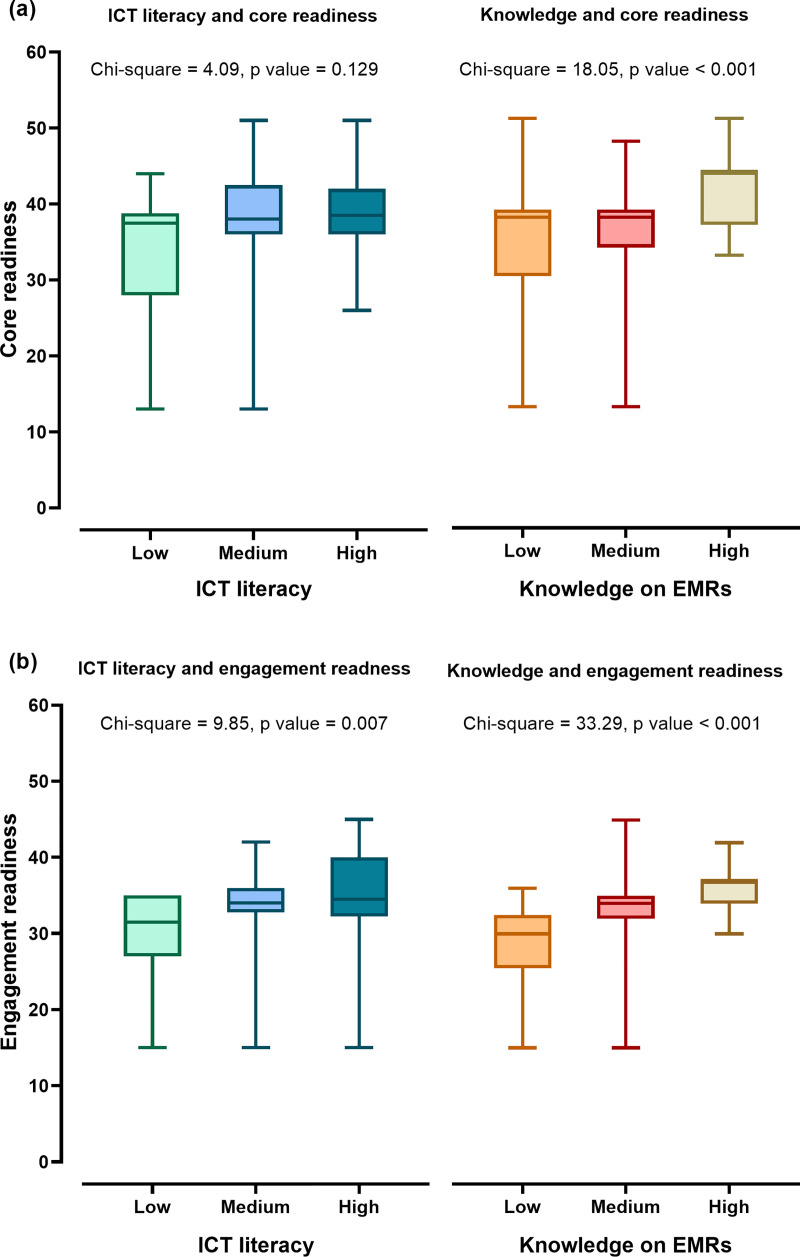
Median (IQR) scores of core and engagement readiness for EMRs adoption at low, medium and high levels of ICT literacy and knowledge on EMRs among health professionals. Box plots referred that core readiness (a) and engagement readiness (b) for EMRs adoption were compared in health professionals with low, medium and high levels of ICT literacy and knowledge on EMRs. (ICT: Information and Communication Technology, EMRs: Electronic Medical Record System, p value by Kruskal-Wallis test).

As shown in (Fig 1), the core readiness scores were significantly different in level of knowledge on EMRs (p value < 0.001). Additionally, there were significantly differences of engagement readiness scores in level of ICT literacy (p value = 0.007) and level of knowledge on EMRs (p value < 0.001). However, there was no statistically difference of core readiness scores in level of ICT literacy (p value = 0.129).

The associated factors of overall readiness for EMRs adoption among health professionals were shown in Table 5. The medical doctors (COR: 2.55, 95% CI: 1.14–5.75), postgraduates (COR: 4.72, 95% CI: 2.07–10.77), and knowledge on EMRs (COR: 1.22, 95% CI: 1.09–1.34) were significant predictors of overall readiness for EMRs adoption. After adjusting the confounding factors, education and knowledge on EMRs were remained as the significant associated factors of overall readiness for EMRs adoption. The postgraduates were 7.32 times more likely to have overall readiness for EMRs adoption than the graduates (AOR: 7.32, 95% CI: 2.26–23.68). An increase of 1 in the knowledge on EMRs score increased the odds of readiness for EMRs adoption over the odds for not readiness by 1.27 times (AOR: 1.27, 95% CI: 1.13–1.43).

**Table 5:** Factors associated with overall readiness for EMRs adoption among health professionals.

Variables			Overall readiness *		p value	COR (95% CI)	p value	AOR (95% CI) ^†^
			Not ready	Ready				
**Socio-demographic Factors**								
	Sex							
		Female	5 (55.6)	4 (44.4)		1.00		1.00
		Male	49 (45.0)	60 (55.0)	0.542	1.53 (0.39–6.01)	0.560	1.65 (0.31–8.83)
	Age		30.00 (8.25, 27.00–35.25)	30.00 (7.75, 28.00–35.75)	0.559	0.98 (0.93–1.04)	0.389	0.89 (0.69–1.16)
	Professional							
		Nurse	42 (53.2)	37 (46.8)		1.00		1.00
		Medical doctor	12 (30.8)	27 (69.2)	0.023	2.55 (1.14–5.75)	0.476	1.65 (0.42–6.47)
	Education							
		Graduate	43 (59.7)	29 (40.3)		1.00		1.00
		Postgraduate	11 (23.9)	35 (76.1)	<0.001	4.72 (2.07–10.77)	0.001	7.32 (2.26–23.68)
	Duration of service		10.00 (7.25, 7.75–15.00)	10.00 (7.00, 7.00–14.00)	0.388	0.98 (0.92–1.03)	0.642	1.07 (0.81–1.40)
	Reported English language skills		4.00 (3.00, 4.00–7.00)	4.00 (4.00, 4.00–8.00)	0.281	1.12 (0.91–1.37)	0.447	0.88 (0.64–1.22)
	ICT literacy		31.00 (16.25, 22.00–38.25)	37.00 (15.00, 24.00–39.00)	0.060	1.04 (0.99–1.07)	0.096	0.95 (0.89–1.01)
	Knowledge on EMRs		11.00 (6.00, 8.00–14.00)	14.00 (6.00, 12.00–18.00)	<0.001	1.22 (1.09–1.34)	<0.001	1.27 (1.13–1.43)

ICT: Information Communication and Technology, EMR: Electronic Medical Record Systems, COR: Crude Odds Ratio, AOR: Adjusted Odds Ratio

* Data were described as n (%) or median (IQR).

^**†**^ All variables in bivariable analysis were included in multivariable regression model. The interaction tests showed no evidence for a difference in the effect of education by knowledge on EMRs (p value < 0.001 for both bivariable and multivariable analysis, likelihood ratio test).

## Discussion

This study explored the ICT literacy, knowledge, and readiness for EMRs adoption among health professionals in a tertiary hospital, Myanmar, that was stepping into the EMRs adoption process. The technology for information and communication in a healthcare setting is vital for modern healthcare systems and effective healthcare services. ICT literacy was the major factor in the acceptance of EMRs and had a positive impact on accessibility to healthcare services, patient satisfaction, effectiveness, and efficiency of healthcare services [26]. Out of 118 health professionals, almost were male, and the male-female ratio was 12:1. It might be due to the differences in demographic background, the nature of professional and types of hospital that provided health care services. In this study, 20.3% of the health professionals had high ICT literacy and it was lower as compared to other studies conducted in India [27], Australia [28], and Ethiopia [29] that reported 75.0%, 70.0%, and 74.3% respectively. Moreover, it was also lower than the previous studies carried out in Ethiopia stated that there were 33.7% of health professionals with adequate knowledge of ICT [30] and 58.7% of health professionals with computer literate [10]. It might be due to the fact that there were differences in sociodemographic characteristics, exposure to ICT of the study populations and cutoff values in score of ICT literacy.

The professional, education, duration of service, and reported English language skills were associated with the ICT literacy among health professionals. The current study stated that the doctors were higher in ICT literacy than the nurses. It could be assumed that doctors were increasingly exposed to ICT in their daily tasks and involved in healthcare delivery by using it. Nonetheless, it was in conflict with the finding of a study conducted in Addis Ababa hospitals, Ethiopia reported that the professional was not associated with ICT literacy [30]. In the finding of this study, the postgraduates were higher in ICT literacy than the graduates. It was inconsistent with a study from Ethiopia [30], which reported that the education of the health workers was not associated with computer knowledge, which may be due to a faster rate of improvement of health information technology in recent years, and widely use of ICT among the health professionals.

Moreover, the health professionals with 6–10 years of service duration were more likely to have high ICT literacy compared with the health professionals with other categories of service duration. It might be due to an association with more widely available in workplaces, the ability to understand the technology, and more exposure to and use of ICT. English languages skill played an important role in the process of developing ICT literacy. In the findings of this study, the health professionals with high English language skills were higher in ICT literacy than those who had low English language skills. On the other hand, sex and age were not statistically significant with the ICT literacy of the study participants. These findings were consistent with a similar study carried out in Ethiopia [30]. However, a study conducted in Australia [28] reported that young health professionals were more frequent users of computers, mobile devices, email, the internet compared with older health professionals. It was concerning as the ICT literacy level of the health professionals was shown to affect the engagement with the health information system.

EMRs could improve the quality of healthcare by reducing the time-consuming in healthcare delivery through enable storage of large data and facilitating quick data retrievals [31]. The knowledge about EMRs of health professionals was also important to increase the adoption and success rate of EMRs [32]. The prevalence of high knowledge on EMRs among health professions was 24.6% and it was lower than the findings of the studies conducted in Ethiopia 45.9% [32], Myanmar 51.0% [20], and Iran 53.6% [33]. Additionally, the studies conducted in Ethiopia reported that 62.6% and 71.3% of the health professionals had high level of knowledge on EMRs [10, 34]. These differences might be attributed to the existing knowledge about EMRs, affordable on training varied in different settings, and variability of categorization in level of knowledge on EMRs.

In this study, the health professionals with more than 10 years of service duration were more likely to have high knowledge on EMRs than other categories of service duration. This finding might be explained by the fact that the health professionals with long duration of service might receive on-the-job training in EMRs and might be more likely willing to use the EMRs in healthcare services. Another possible explanation for this result was that they might be more aware of the challenges of paper-based medical record system such as lack of storage space, medical errors, patient’s safety and data confidentiality. A study carried out among the healthcare workers in Nigeria reported that the duration of services was not associated with the knowledge on EMRs [35].

EMRs assisted in providing better evidence-based healthcare, validity, and accessibility of patient information, safer prescribing, better clinical decision, and improved medical practice efficiency [36–39]. Consequently, it is important to address the readiness for EMRs adoption in health professionals in order to observe the benefits of adoption [40]. In this study, the overall readiness for EMRs adoption in health professionals was 54.2% with core readiness 59.3% and engagement readiness 61.9%. In contrast to other studies, overall health professionals’ readiness was higher than the study conducted in Myanmar (47.1%, with core readiness 62.2% and engagement readiness 63.0%) [20]. This result seemed to be consistent with other studies done in Ethiopia (54.1%, with core readiness 67.8% and engagement readiness 60.9%) [10], Ghana (54.9%, with core readiness 67.2% and engagement readiness 43.1%) [41] and Iran (56.0%) [33]. However, it was lower than the previous another study conducted in Ethiopia (62.3%, with core readiness 66.2% and engagement readiness 65.2%) [34]. The differences could be explained that the readiness of health professionals could be attributed to technological development in the past few years, the high ICT literacy and level of knowledge about EMRs, expressing the dissatisfaction with the paper record system, and willingness for adoption. In addition, other possible explanations were that this discrepancy could be due to variation in sample size, differences in utilization of tools applied for the assessment of readiness for EMRs adoption, and variability of cutoff value in scoring system.

In this study, the postgraduates were more likely to be ready for EMRs adoption than the graduates. This is a logical finding that health professionals with higher educational levels might have more ability to afford to learn ICT and willingness to adopt the EMRs for an effective healthcare system. This observed association persisted after adjusting the confounding variables, however, a similar study conducted in northern Ghana showed that the education of the health professionals was not associated with readiness for EMRs adoption [41]. This might be associated with the differences in sociodemographic background and the rate of distribution of ICT accessibility in the study population.

The health professionals with high knowledge on EMRs were more likely to have readiness for EMRs adoption than those who had low knowledge on EMRs. This could be expected that the health professionals with high knowledge on EMRs might have more utilization of computer which was related to familiarity with the ICT and more interest in the adoption of the system. This finding remained as a significant predictor after performing multivariable logistic regression analysis, and it was consistently in line with the studies conducted in Ethiopia [10, 34], Myanmar [20] and Ghana [41] showed that the knowledge on EMRs was associated with the health professionals’ readiness for EMRs adoption.

On the other hand, sex, age, professional, duration of service, reported English language skills, and ICT literacy of health professionals were not associated with readiness for EMRs adoption. However, similar studies conducted in Ethiopia [34] and Ghana [41] reported that computer literacy of participants was associated with the health professionals’ readiness for EMRs adoption, and then another Ethiopia study [10] also showed that sex and computer literacy had a significant association with health professionals’ readiness for EMRs adoption.

### Strength and limitation

This descriptive study could assess the readiness for EMRs adoption among the health professionals in the tertiary hospital before implementation process. There were some limitations to this study. The reported English language skills and ICT literacy of the health professionals were provided by the subjective assessment. Further studies should be conducted for an objective assessment of the English language skill and ICT literacy to determine the training programs that were designed for improving the English language and IT skills of health professionals. This study only assessed the core and engagement readiness, and therefore the technology and societal readiness of the health professionals should be assessed by further studies. Although the findings of this study may not be generalizable to the study population of other countries, it was generalized to the health professionals who have similar demographics in other tertiary hospitals that are implementing the EMRs adoption. Moreover, the findings of this study highlighted the importance of improving ICT literacy and readiness assessment for EMRs adoption in health professionals in developing countries.

## Conclusions

The prevalence of high level of ICT literacy and knowledge on EMRs of health professionals in the study area were 20.3% and 24.6% respectively. The factors associated with ICT literacy were the professional, education, duration of service, and reported English language skills. It is recommended that enhancing the accessibility of computers and providing the training program related to ICT development skills and English language skills should be needed for the improvement of ICT literacy, computer utilization, and diffusion rate of ICT in the healthcare organization. Duration of service was an associated factor of knowledge on EMRs and encouragement of EMRs training program enabling hands-on experience for the health professional. The overall readiness of health professionals for EMRs adoption was 54.2% in this study area. Education and knowledge on EMRs were significant predictors of health professionals’ readiness for EMRs adoption. Contextualization and scaling up of the curriculum-related EMRs are important for the development of successful EMRs in a healthcare setting. Additionally, enhancing the EMRs training to health professionals involving all levels of adoption stages should be carried out to increase the willingness to accept and readiness for EMRs adoption.

## Supporting information

S1 File English version of the questionnaire. (PDF)

S2 File Myanmar version of the questionnaire. (PDF)

S3 File Scoring system. (PDF)

S1 Data Minimal data. (XLSX)
