# Supplementary material for: Information and communication technology literacy, knowledge and readiness for electronic medical record system adoption among health professionals in a tertiary hospital, Myanmar: A cross-sectional study
Source: PLoS One. 2021 Jul 1;16(7):e0253691. doi: 10.1371/journal.pone.0253691 (PMC8248629; doi:10.1371/journal.pone.0253691)
Supplement: S2 File — (PDF) [file pone.0253691.s002.pdf]

မေးခွန်းလွှာ

ကုဒ်နံပါတ် ☐☐☐

ပုဂ္ဂိုလ်ရေးဆိုင်ရာအချက်အလက်များ				
အသက် (ပြည့်ပြီး)	နှစ်		☐ ကျား	☐ မ
အလုပ်အကိုင်			☐ ဆရာဝန်	☐ သူနာပြု
ပညာရေးအဆင့်	☐ Bachelor	☐ Diploma	☐ Master	☐ Ph.D./Doctorate
စုစုပေါင်းလုပ်သက်				နှစ်

**အင်္ဂလိပ်စာကျွမ်းကျင်မှု**			
	**အခြေခံ**	**အလယ်အလတ်**	**ကျွမ်းကျင်**
Reading	☐	☐	☐
Writing	☐	☐	☐
Listening	☐	☐	☐
Speaking	☐	☐	☐

**သတင်းအချက်အလက်၊ ဆက်သွယ်မှုနည်းပညာနှင့် ပတ်သတ်သော အသိပညာဗဟုသုတ**			
၁	ကွန်ပျူတာအသုံးပြုဖူးပါသလား။ (အကယ်၍ အသုံးမပြုဖူးပါက မေးခွန်းနံပါတ် (၈)သို့ကျော်ရန်)	☐ အသုံးပြုဖူးပါသည်။	☐ အသုံးမပြုဖူးပါ။
၂	သင့်အိမ်/အဆောင်/အခန်းမှာ ကွန်ပျူတာကိုယ်ပိုင် ရှိပါသလား။	☐ ရှိပါသည်။	☐ မရှိပါ။
၃	အလုပ်မှာ ကွန်ပျူတာအသုံးပြုရန် အသင့်ရှိပါသလား။	☐ ရှိပါသည်။	☐ မရှိပါ။
၄	ကွန်ပျူတာအသုံးပြုသည့်အကြောင်းအရင်း		
	အလုပ်အတွက်	☐ သုံးပါသည်။	☐ မသုံးပါ။
	ပညာရေးအတွက်	☐ သုံးပါသည်။	☐ မသုံးပါ။
	အဆက်အသွယ်ပြုလုပ်ရန်အတွက်	☐ သုံးပါသည်။	☐ မသုံးပါ။
	အပန်းဖြေရေးအတွက်	☐ သုံးပါသည်။	☐ မသုံးပါ။
	ကစားရန်အတွက်	☐ သုံးပါသည်။	☐ မသုံးပါ။
၅	ကွန်ပျူတာနှင့်ပတ်သက်၍ သင်တန်းတက်ဖူးပါသလား။	☐ တက်ဖူးပါသည်။	☐ မတက်ဖူးပါ။
၆	ကွန်ပျူတာကျွမ်းကျင်မှုနှင့်ပတ်သက်လို့ သင့်ကိုယ်သင် ဘယ်လိုယူဆပါသလဲ။		
	☐ ကွန်ပျူတာအကြောင်း လုံးဝမသိပါ။		

☐ ကွန်ပျူတာအကြောင်း အတော်အသင့်သိပါသည်။

☐ ကွန်ပျူတာအသုံးပြုဖို့အတွက် ကိုယ့်ကိုယ်ကို ယုံကြည်မှုအပြည့်ရှိပါသည်။

၇ သင့်၏ကွန်ပျူတာမှာ စာရိုက်ကျွမ်းကျင်မှုနှင့်ပတ်သက်လို့ ဘယ်လိုထင်သလဲ။

☐ စာ ဘယ်လိုရိုက်ရမှန်းမသိပါ။

☐ စာတော့ရိုက်တတ်တယ် ကျွမ်းကျွမ်းကျင်ကျင်တော့မဟုတ်ပါ။

☐ စာရိုက်တာကျွမ်းကျင်တယ်လို့ ယုံကြည်မှုအပြည့်ရှိပါတယ်။

၈ Smartphone/Tablet device ကိုလအသုံးပြုပါ သလား။ ☐ အသုံးပြုပါသည်။ ☐ အသုံးမပြုပါ။

၉ ကွန်ပျူတာနှင့်ဆက်စပ်ပစ္စည်းများ/Smartphone/Tablet device တွေ အသုံးပြု နိုင်ဖို့ ဘယ်လို သင်ယူပါသလဲ။

သူငယ်ချင်းများထံမှ	☐ သင်ယူပါသည်။	☐ မသင်ယူပါ။
မိသားစုဝင်များထံမှ	☐ သင်ယူပါသည်။	☐ မသင်ယူပါ။
သင်တန်းတက်ခြင်းဖြင့်	☐ သင်ယူပါသည်။	☐ မသင်ယူပါ။
အင်တာနက်မှ	☐ သင်ယူပါသည်။	☐ မသင်ယူပါ။

၁၀

ကွန်ပျူတာ/mobile applications တွေကို အသုံးပြုတတ်ခြင်း။	**မသုံးတတ်**	**အခြေခံ**	**အလယ်အလတ်**	**ကျွမ်းကျင်**
Microsoft Word	☐	☐	☐	☐
Microsoft Excel	☐	☐	☐	☐
Microsoft PowerPoint	☐	☐	☐	☐
Database (Access)	☐	☐	☐	☐
Photo editing	☐	☐	☐	☐
Internet	☐	☐	☐	☐
Email	☐	☐	☐	☐
Facebook	☐	☐	☐	☐
WhatsApp	☐	☐	☐	☐

၁၁ သင်၏ Smartphone/Tablet တွေမှာ စာရိုက်တာနှင့်ပတ်သက်လို့ ကိုယ့်ကိုယ်ကို ဘယ်လိုထင်ပါသလဲ။

☐ စာ ဘယ်လိုရိုက်ရမှန်းမသိပါ။

☐ စာတော့ရိုက်တတ်တယ် ကျွမ်းကျွမ်းကျင်ကျင်တော့မဟုတ်ပါ။

☐ စာရိုက်တာကျွမ်းကျင်တယ်လို့ ယုံကြည်မှုအပြည့်ရှိပါတယ်။

၁၂ သင်၏ကွန်ပျူတာကျွမ်းကျင်မှုနှင့် ဗဟုသုတအကြောင်းဖော်ပြပါ။

ကွန်ပျူတာ Power အဖွင့်/အပိတ် လုပ်တတ်ပါသည်။	☐ အသုံးပြုပါသည်။	☐ အသုံးမပြုပါ။
Mouse/Track pad အသုံးပြုတတ်ပါသည်။	☐ အသုံးပြုပါသည်။	☐ အသုံးမပြုပါ။
USB flash drive (memory stick/SD card) ကို format ချတတ်ပါသည်။	☐ အသုံးပြုပါသည်။	☐ အသုံးမပြုပါ။
USB flash drive (memory stick/SD card) ထဲမှာ data သိမ်းတတ်ပါသည်။	☐ အသုံးပြုပါသည်။	☐ အသုံးမပြုပါ။
Application တစ်ခုမှ တစ်ခုသို့ စာတွေ/ပုံတွေကို copy/paste လုပ်တတ် ပါသည်။	☐ အသုံးပြုပါသည်။	☐ အသုံးမပြုပါ။
စာရွက်မှတ်တမ်းတစ်ခုကို Print ထုတ်တတ်ပါသည်။	☐ အသုံးပြုပါသည်။	☐ အသုံးမပြုပါ။
Folder/File directory များ တည်ဆောက်တတ်ပါသည်။	☐ အသုံးပြုပါသည်။	☐ အသုံးမပြုပါ။
Essay/Letter/CV Form တစ်ခုကို မိမိဘာသာ ပြုလုပ်ဖန်တီးတတ်ပါသည်။	☐ အသုံးပြုပါသည်။	☐ အသုံးမပြုပါ။
ဖုန်း(သို့မဟုတ်)ကွန်ပျူတာ Application အသစ်တစ်ခုကို သုံးစွဲဖို့ မိမိဘာသာ လေ့လာသင်ယူနိုင်သည်။	☐ အသုံးပြုပါသည်။	☐ အသုံးမပြုပါ။
Email အသုံးပြုတတ်ပါသည်။	☐ အသုံးပြုပါသည်။	☐ အသုံးမပြုပါ။
Email တွင် file attachment ထည့်သွင်းပို့တတ်ပါသည်။	☐ အသုံးပြုပါသည်။	☐ အသုံးမပြုပါ။
Mailbox စာ အဝင်/အထွက်ပုံးများကိုစီစဉ်တတ်ပါသည်။	☐ အသုံးပြုပါသည်။	☐ အသုံးမပြုပါ။
File format တွေနဲ့ပတ်သက်လို့ နားလည်ပါသည်။ (ဥပမာ - pdf, doc, jpg).	☐ အသုံးပြုပါသည်။	☐ အသုံးမပြုပါ။

**ကွန်ပျူတာဆေးမှတ်တမ်းထိန်းစနစ်နှင့် ပတ်သတ်၍ အသိပညာဗဟုသုတ**			
၁	ကွန်ပျူတာဆေးမှတ်တမ်းထိန်းစနစ်အကြောင်း သင်မည်မျှသိပါသနည်း။		
	☐ လုံးဝမသိပါ။		
	☐ အနည်းငယ်မျှသာသိပါသည်။		
	☐ အနည်းအကျဉ်းသိပါသည်။		
	☐ အတော်အသင့်သိပါသည်။		
	☐ တော်တော်များများသိပါသည်။		
၂	ကွန်ပျူတာဆေးမှတ်တမ်းထိန်းစနစ် (EMR system) သည် ရုံးလုပ်ငန်းသုံး အတွက်သာ အသုံးဝင်ပြီး လူနာဆေးကုသမှုအတွက် အသုံးမဝင်ပါ။		
	☐ မှန်	☐ မှား	☐ မသိပါ

၃	ကွန်ပျူတာဆေးမှတ်တမ်းထိန်းစနစ် (EMR system) သည် အတွင်းလူနာနှင့် ပြင်ပလူနာအတွက်ပါ အသုံးပြုနိုင်သည်။<br>☐ မှန် ☐ မှား ☐ မသိပါ
၄	ကွန်ပျူတာသုံး ဆေးမှတ်တမ်းနည်းပညာ(EMR technology)နှင့် ဆေးပစ္စည်း ကိရိယာများ ချိတ်ဆက်အသုံးပြုခြင်းဖြင့် အချိန်ကုန်သက်သာစေသည့်အပြင် အောက်ပါတို့ကို လျှော့ကျစေနိုင်သည်။<br>☐ သွေးများပေကျံခြင်း ☐ အဖြေကူးယူမှုလွဲမှားခြင်း ☐ ဓာတ်ခွဲစမ်းသပ်မှုများ ထပ်လုပ်မိခြင်း ☐ မသိပါ
၅	ကွန်ပျူတာဆေးမှတ်တမ်းထိန်းစနစ် (EMR system) အကောင်အထည်ဖော်ဖို့ အဓိက အခက်အခဲ/ အတားအဆီးများမှာ-
☐	လူနာ၏အချက်အလက်များတူညီနေခြင်း
☐	စံသက်မှတ်ထားသော ပုံသေအသုံးအနှုန်းများမရှိခြင်း
☐	ကွန်ပျူတာတတ်ကျွမ်းနားလည်မှုမရှိခြင်း
☐	ပြောင်းလဲရန်အတွက် ခုခံမှုများရှိခြင်း
☐	ငွေကြေးထောက်ပံ့မှု ခက်ခဲခြင်း
☐	လိုအပ်သော အချက်အလက်ရရှိရန် ကျန်းမာရေးဝန်ထမ်းများထံမှ တောင်းခံရခြင်း
☐	လူနာ၏အချက်အလက်များ လုံခြုံစွာထိန်းသိမ်းထားရခြင်း
☐	အချက်အလက်များတိကျမှုရှိရန်ထိန်းသိမ်းရခြင်း
☐	ရောဂါအမျိုးအစားခွဲခြားမှု အားနည်းခြင်း
☐	ဝန်ထမ်းများ ကျွမ်းကျင်မှုအားနည်းခြင်း
☐	လုပ်ငန်းခွင်ပတ်ဝန်းကျင် ပြဿနာများရှိခြင်း
☐	ဆရာဝန်များနှင့် အုပ်ချုပ်မှုပိုင်းဆိုင်ရာတာဝန်ခံများ ပါဝင်မှုအားနည်းခြင်း
၆	ကွန်ပျူတာဆေးမှတ်တမ်းထိန်းစနစ် (EMR system) ဆိုတာ လူနာမှတ်တမ်း များကို စာရွက်ပေါ်တွင် ရေးမှတ်ခြင်းအစား ကွန်ပျူတာနှင့် စမတ်ဖုန်း/တက်ဘလက် များပေါ်မှာ ရေးမှတ်ခြင်းပင်ဖြစ်သည်။ တခြားထူးခြားတာမရှိပါ။<br>☐ မှန် ☐ မှား ☐ မသိပါ
၇	ရောဂါနှင့်ပတ်သက်သည့် လူနာပြောကြားချက်များကို ကွန်ပျူတာ ဆေးမှတ်တမ်းထိန်းစနစ်တွင် ထည့်သွင်းမဖော်ပြပါ။<br>☐ မှန် ☐ မှား ☐ မသိပါ
၈	Digital နည်းပညာဖြင့် ဓာတ်မှန်အဖြေများသိမ်းဆည်းခြင်းဖြင့် ဓာတ်မှန်ဌာနတွင် ကုန်ကျစရိတ် သက်သာစေသောအကြောင်းအရင်းမှာ-<br>☐ ဓာတ်မှန်ဌာနတွင် နေရာပိုများရရှိလာခြင်း<br>☐ ဓာတ်မှန်ဖလင်နှင့် စာရွက်အတွက် ကုန်ကျစရိတ်မရှိခြင်း<br>☐ ဝန်ထမ်းများ၏စွမ်းဆောင်ရည်တက်လာခြင်း<br>☐ အထက်ပါအားလုံး

	☐ အထက်ပါအားလုံး တစ်ခုမှမမှန်ပါ<br>☐ မသိပါ
၉	ကွန်ပျူတာဆေးမှတ်တမ်းထိန်းစနစ် အသုံးပြုပြီးနောက်ပိုင်းတွင် ခာတ်မှန်နှင့် ခာတ်ခွဲစမ်းသပ်ချက်များ တောင်းခံသည့်အခါ လူနာ၏အချက်အလက်များ- (ဥပမာ - အမည်၊ အသက်၊ ကျား/မ) ကို ကွန်ပျူတာမှ စာရွက်ပေါ်သို့ ကူးယူရေးသားဖို့ လိုအပ်ပါသည်။<br>☐ မှန် ☐ မှား ☐ မသိပါ
၁၀	ကွန်ပျူတာဆေးမှတ်တမ်းထိန်းစနစ် အသုံးပြုသည့်အခါ လူနာမှတ်တမ်း များအား ဖောက်ထွင်း ခိုးယူမည့်အန္တရာယ် ရှိပါသည်။<br>☐ မှန် ☐ မှား ☐ မသိပါ
၁၁	ICD-10 code ဆိုသည်မှာ မည်သည့်အကြောင်းအရာတွင် ထားရှိသည့် code စနစ် ဖြစ်သနည်း။<br>☐ ဆေးရုံ ☐ ရောဂါ ☐ ဆေးဝါး ☐ မသိပါ
၁၂	ကွန်ပျူတာဆေးမှတ်တမ်းထိန်းစနစ် သည် ဆရာဝန်များအား လူနာအတွက် ဆေးဝါးကုသမှုနှင့် ပတ်သက်၍ ဆုံးဖြတ်ချက်ချနိုင်ရန် အထောက်အကူ ပေးနိုင် ပါသည်။<br>☐ မှန် ☐ မှား ☐ မသိပါ
၁၃	ကွန်ပျူတာဆေးမှတ်တမ်းထိန်းစနစ် (EMR system) သည် လူနာအား ဆေးရုံ အလွှဲ/ အပြောင်း၊ လူနာဆောင် အလွှဲ/အပြောင်းအတွက် အသုံးဝင်ပါသည်။<br>☐ မှန် ☐ မှား ☐ မသိပါ
၁၄	ကွန်ပျူတာဆေးမှတ်တမ်းထိန်းစနစ် (EMR system) နှင့် ခာတ်ခွဲခန်း အချက်အလက် စနစ်များကို အသုံးပြုခြင်းဖြင့် ခာတ်ခွဲစမ်းသပ်ချက်အဖြေများကို ကွန်ပျူတာထဲတွင် သိမ်းဆည်းထားပြီး လူနာဆောင်မှ ဆရာဝန်က တိုက်ရိုက်ကြည့်ရှု နိုင်မည်ဖြစ်သည်။<br>☐ မှန် ☐ မှား ☐ မသိပါ

ကွန်ပျူတာဆေးမှတ်တမ်းထိန်းစနစ်အား မူလအတွင်းစိတ် အားဖြင့် အဆင်သင့်ဖြစ်မှု					
	**မေးခွန်း**	လုံးဝသဘောမတူပါ	သဘောမတူပါ	သဘောတူပါသည်	လုံးဝသဘောတူပါသည်
**မှတ်တမ်းမှတ်ရာများ ထိရောက်မှုရှိ/မရှိ**					
၁	လက်ရှိအသုံးပြုနေသော လူနာမှတ်တမ်းစနစ်သည် စာရွက် စာတမ်းအလုပ်များသာ များလွန်းပါသည်။	☐	☐	☐	☐

၂	စာရွက်ဆေးမှတ်တမ်းများသည် သိမ်းဆည်းရာတွင် နေရာအလွန်ယူသဖြင့် ထိန်းသိမ်းရခက်ပါသည်။	☐	☐	☐	☐
၃	စာရွက်ပေါ်ရှိ ဆေးမှတ်တမ်းများအား လိုအပ်သည့်အချိန် ပြန်ရှာရန်တွက် အခက်အခဲရှိပါသည်။	☐	☐	☐	☐
၄	လက်ရှိလူနာဆေးမှတ်တမ်းများ ရေးသွင်းအသုံးပြုရတာ အချိန်အရမ်းကုန်ပါသည်။	☐	☐	☐	☐
၅	လက်ရှိ စာရွက်အသုံးပြုနေသောဆေးမှတ်တမ်းစနစ်သည် လုပ်အား များများလိုအပ်ပါသည်။	☐	☐	☐	☐
**လူနာအချက်အလက်ပေါက်ကြားမှု ရှိ/မရှိ**					
၆	လူနာမှတ်တမ်း (Patient chart) များသည် လူနာအချက်အလက်များလုံခြုံရေးအတွက် စိတ်မချရပါ။	☐	☐	☐	☐
၇	လက်ရှိအသုံးပြုနေသော လူနာဆေးမှတ်တမ်းများသည် ခိုးယူ/ပျောက်ဆုံးမှုရှိနိုင်သည်။	☐	☐	☐	☐
၈	လက်ရှိအသုံးပြုနေသောဆေးမှတ်တမ်းစနစ်သည် လူနာမှတ်တမ်း/အချက်အလက် လုံခြုံရေးအတွက် အကောင်းဆုံးဖြစ်သည်။	☐	☐	☐	☐
**အချက်အလက်ပြည့်စုံမှုနှင့် တိကျမှုအတွက် စိတ်ကျေနပ်မှု ရှိ/မရှိ**					
၉	ဆေးမှတ်တမ်းစာရွက်များသည် ပျက်စီးလွယ်သောကြောင့် အချက်အလက်များ ဆုံးရှုံးမှုရှိနိုင်ပါသည်။	☐	☐	☐	☐
၁၀	လူနာဆေးမှတ်တမ်းပေါ်ရှိ လက်ရေးလက်သားမရှင်းလင်းမှုများကြောင့် ဆေးဝါးကုသမှုတွင် အမှားအယွင်းများဖြစ်ပေါ် ပါသည်။	☐	☐	☐	☐
၁၁	လက်ရှိဆေးမှတ်တမ်းစနစ် အားနည်းချက်ကြောင့် ဆေးကုသရာတွင် ဆေးဝါးလွဲမှားခြင်း၊ ရောဂါအမည်တတ် (Diagnosis) လွဲမှားခြင်းများဖြစ်ပေါ်နိုင်သည်။	☐	☐	☐	☐
**လူနာမှတ်တမ်းများ မျှဝေသုံးစွဲမှုအတွက် အခက်အခဲများ**					
၁၂	လက်ရှိအသုံးပြုနေသော ဆေးမှတ်တမ်းစနစ်သည် လူနာအချက်အလက်ပေးပို့မှုအပိုင်းတွင် နှေးကွေးသည်ဟု ခံစားမိပါသည်။	☐	☐	☐	☐
၁၃	စာရွက်မှတ်တမ်းစနစ် အားနည်းချက်များကြောင့် ဓာတ်ခွဲ/ဓာတ်မှတ်စမ်းသပ်မှုများ ထပ်ခါတစ်လဲလဲလုပ်မိနိုင်ပါသည်။	☐	☐	☐	☐

ကွန်ပျူတာသုံးဆေးမှတ်တမ်းထိန်းစနစ်အား ထိပ်တိုက်တွေ့ဆုံရန် အဆင်သင့်ဖြစ်မှု					
	မေးခွန်း	လုံး၀သဘောမတူပါ	သဘောမတူပါ	သဘောတူပါသည်	လုံး၀သဘောတူပါသည်
**ဖြစ်လာနိုင်သော ဆိုးကျိုးသက်ရောက်မှုများ**					
၁	ကွန်ပျူတာသုံးဆေးမှတ်တမ်းစနစ်အသုံးပြုနိုင်ရန်အတွက် ကုန်ကျစရိတ်မြင့်မားသဖြင့် လက်ရှိအသုံးပြုနေသော စာရွက်စာတမ်းစနစ်အား မပြောင်းလဲသင့်ပါ။	☐	☐	☐	☐
၂	မိမိတွင် ကွန်ပျူတာဗဟုသုတမရှိသောကြောင့် ကွန်ပျူတာ စနစ်ထက် လက်ရှိအသုံးပြုနေသော ဆေးမှတ်တမ်းစနစ် ကို ပိုအားရပါသည်။	☐	☐	☐	☐
၃	ကွန်ပျူတာသုံးဆေးမှတ်တမ်းစနစ်ကို အပြည့်အ၀ အသုံးပြု နိုင်ရန် အချိန်များစွာလိုအပ်ပြီး အကျိုးသိပ်မရှိနိုင်ပါ။	☐	☐	☐	☐
၄	ကွန်ပျူတာစနစ်ပြောင်းလဲခြင်းကြောင့် အထစ်အငေါ့မရှိ သော လက်ရှိလုပ်ငန်းစီးဆင်းမှုများ ထိခိုက်မည်ကို စိုးရိမ်မိပါသည်။	☐	☐	☐	☐
**ကောင်းကျိုးများကို သိမြင်ခြင်း**					
၅	ကွန်ပျူတာဆေးမှတ်တမ်းစနစ် အသုံးပြုခြင်းဖြင့် လူနာ စောင့်ရှောက်ခြင်းအတွက် ပိုမိုထိရောက်စေပါသည်။	☐	☐	☐	☐
၆	ကွန်ပျူတာဆေးမှတ်တမ်းစနစ်သည် လူနာ၏ ကိုယ်ရေး ကိုယ်တာမှတ်တမ်းများ လုံခြုံရေးအတွက် ပို၍စိတ်ချရပါသည်။	☐	☐	☐	☐
၇	ကွန်ပျူတာဆေးမှတ်တမ်းစနစ်အသုံးပြုခြင်းဖြင့် လူနာများ ဆေးကုသမှုအတွက် စောင့်ဆိုင်းရချိန်များ လျော့နည်းစေပါသည်။	☐	☐	☐	☐
၈	ကွန်ပျူတာဆေးမှတ်တမ်းစနစ်အသုံးပြုခြင်းဖြင့် ဆရာ၀န်/ သူနာပြုများ အချင်းချင်း၊ ဆေးရုံအချင်းချင်း၊ လူနာမှတ်တမ်း များ ပိုမိုကောင်းမွန်စွာ မျှဝေသုံးစွဲနိုင်ပါမည်။	☐	☐	☐	☐
၉	ကွန်ပျူတာဆေးမှတ်တမ်းစနစ် ပြောင်းလဲခြင်းသည် စာရွက် ပေါ် တွင် ဘောပင်နှင့်ရေးသားခြင်းအစား ကွန်ပျူတာ ကီးဘုတ်ဖြင့် ရေးသားခြင်းသာကွာခြားပြီး ဘာမှ ပိုအကျိုးမရှိ နိုင်ပါ။	☐	☐	☐	☐
**ကွန်ပျူတာဆေးမှတ်တမ်းစနစ် (EMR system) ပြောင်းလဲရန် ဆန္ဒ ရှိ/မရှိ**					
၁၀	ကွန်ပျူတာဆေးမှတ်တမ်းစနစ် ပြောင်းလဲရန် မိမိမသိရှိ သေးသည်များကို လေ့လာသင်ယူရန် ဆန္ဒရှိပါသည်။	☐	☐	☐	☐

၁၁	မိမိသည် ကွန်ပျူတာဗဟုသုတနည်းပါးသဖြင့် ကွန်ပျူတာ ဆေးမှတ်တမ်းထိန်းစနစ်ကိုပြောင်းလဲရမှာကြောက်ပါသည်။	☐	☐	☐	☐
၁၂	ကွန်ပျူတာဆေးမှတ်တမ်းစနစ် ဖြစ်ထွန်းဖို့အတွက် အခွင့် အရေးရှိပါက ကိုယ်တိုင်ပါဝင်ဆောင်ရွက်ချင်စိတ်ရှိပါသည်။	☐	☐	☐	☐
