# Supplementary material for: Information and communication technology literacy, knowledge and readiness for electronic medical record system adoption among health professionals in a tertiary hospital, Myanmar: A cross-sectional study
Source: PLoS One. 2021 Jul 1;16(7):e0253691. doi: 10.1371/journal.pone.0253691 (PMC8248629; doi:10.1371/journal.pone.0253691)
Supplement: S3 File — (PDF) [file pone.0253691.s003.pdf]

Scoring System

Reported English language skills			
	**Basic**	**Intermediate**	**Advanced**
Reading	1	2	3
Writing	1	2	3
Listening	1	2	3
Speaking	1	2	3

**Information and Communication Technology literacy**			
1	Have you ever used a computer? If No, please go question 8.	1 Yes	0 No
2	Do you have your owned/accessible computer at home or residence or hostel?	1 Yes	0 No
3	Do you have any access to computer at work?	1 Yes	0 No
4	What is the purpose of you use a computer? (can choose MULTIPLE answer)		
	Work	1 Yes	0 No
	Education	1 Yes	0 No
	Communication with other people	1 Yes	0 No
	Entertainment	1 Yes	0 No
	Playing games	1 Yes	0 No
5	Did you have any experience with training course about computer and IT?	1 Yes	0 No
6	Which of these statements best describes the way you feel about computers? (Please tick ONE)		
	0 I am completely lacking in confidence		
	1 I feel I can cope		
	2 I feel very confident using computers		
7	How would you describe your typing skills on computer? (Please tick ONE)		
	0 I am completely unfamiliar with the basic of typing		
	1 I can type but have difficulties on some unfamiliar keys		
	2 I am very competent with typing		
8	Do you use smartphone or tablet device?	1 Yes	0 No
9	To use computer, accessories or ICT materials, including smartphones and tablet device, I learn it (can choose MULTIPLE answer)		

	By asking friends			1 Yes	0 No
	By asking family members			1 Yes	0 No
	By taking a training course			1 Yes	0 No
	By learning from online			1 Yes	0 No
10	Use of computer or mobile application	**None**	**Basic**	**Intermediate**	**Advanced**
	Microsoft Word	0	1	2	3
	Microsoft Excel	0	1	2	3
	Microsoft PowerPoint	0	1	2	3
	Database (Access)	0	1	2	3
	Photo editing	0	1	2	3
	Internet	0	1	2	3
	Email	0	1	2	3
	Facebook	0	1	2	3
	WhatsApp	0	1	2	3
11	How would you describe your typing skill on smartphone or mobile tablets? (Please tick ONE)				
	0 I am completely unfamiliar with the basic of typing				
	1 I can type but have difficulties on some unfamiliar keys				
	2 I am very competent with typing				
12	Please indicate which of these skills and knowledge you possess. (can choose MULTIPLE answer)				
	I know how to turn a computer on and off.			1 Yes	0 No
	I am able to use a mouse/track pad.			1 Yes	0 No
	I am able to format a USB flash drive (memory stick/SD card).			1 Yes	0 No
	I know how to save data to a USB flash drive (memory stick/SD card).			1 Yes	0 No
	I can copy and paste information from one application to another.			1 Yes	0 No
	I can print out a document.			1 Yes	0 No
	I can set up folders or file directories.			1 Yes	0 No
	I am able to word process an essay or a letter or my CV.			1 Yes	0 No
	I feel able to teach myself how to use a new application (phone/computer).			1 Yes	0 No
	I can send an email.			1 Yes	0 No
	I can send a file as an email attachment.			1 Yes	0 No
	I can manage mailboxes.			1 Yes	0 No

I understand different file formats (e.g. pdf, doc, jpg).	1 Yes	0 No

Knowledge on electronic medical record system	
1	How much do you know about EMR system?<br>0 None<br>1 Very little<br>2 A few things<br>3 Moderate<br>4 A great deal
2	EMR is only for office use and not applicable for clinical practice.<br>0 Yes 1 No 0 Don't know
3	EMR system is applicable for both inpatient care and outpatient department.<br>1 Yes 0 No 0 Don't know
4	Medical devices which connect to EMR technology will save time and what does reduce opportunities for?<br>0 Contamination of blood 1 Transcription error 0 Repeated tests 0 Don't know
5	What are the important barriers for successful implementation of EMR system (1 point for each)<br>1 Unique patient identification<br>1 Lack of standard terminology<br>1 Lack of computer literacy or technology<br>1 Issue of strong resistance to change<br>1 Financial limitation<br>1 Concern by providers for information available on request<br>1 Confidentiality<br>1 Quality and accuracy of data<br>1 Lack of disease classification<br>1 Lack of staff with adequate skill<br>1 Environmental issue<br>1 Involvement of clinicians and administrator
6	Electronic Medical Record (EMR) system is nothing but writing patient records on computer or smart device rather than on paper.<br>0 Yes 1 No 0 Don't know
7	Patient's complaints are not documented in EMR system.

		0 Yes	1 No	0 Don't know
8	Digital storage results in a significant cost reduction for the imaging center due to which of the following?<br>0 Increase in available space at the facility<br>1 Elimination of films and paper<br>0 Increased efficiency for the staff<br>0 All of the above<br>0 None of the above<br>0 Don't know			
9	After application of EMR system, it is necessary to copy patient information like name, age and sex from computer to paper requisition form while doing laboratory and radiological investigations.			
		0 Yes	1 No	0 Don't know
10	There is risk of patient information being hacked or stolen with the application of EMR.			
		1 Yes	0 No	0 Don't know
11	ICD codes are the standard codes for-			
	0 Hospital	1 Diseases	0 Drugs	0 Don't know
12	EMR system can help clinician in making decision enhancing patient care.			
		1 Yes	0 No	0 Don't know
13	EMR is useful while transferring patient from one ward to another in a hospital as well as form one hospital to another.			
		1 Yes	0 No	0 Don't know
14	By using EMR and Laboratory Information system with automated machines, the investigation results are automatically stored in the computer and can be directly seen by the doctor from patient ward.			
		1 Yes	0 No	0 Don't know

**Core readiness for EMRs adoption**					
	**Statements**	**Strongly disagree**	**Disagree**	**Agree**	**Strongly agree**
**Inefficient documentation**					
1	Currently running manual medical record system is too many paper works.	1	2	3	4
2	Paper based system requires too many spaces and there is difficulty for storage of records.	1	2	3	4

3	Paper based medical records are too difficult to retrieve a record at the time of need.	1	2	3	4
4	Current paper based medical record system is wasting of time.	1	2	3	4
5	Current paper based medical record system is labor intensive.	1	2	3	4
**Breached patient privacy**					
6	I worry about the privacy of patient's information in paper based medical record (Patient chart)	1	2	3	4
7	Patients' records in paper based manual system may be lost or stolen.	1	2	3	4
8	I feel that current paper based medical record system is the best way of securing patient's information and privacy.	4	3	2	1
**Dissatisfaction with completeness and accuracy**					
9	Paper medical records are easy to damage and there can be information loss.	1	2	3	4
10	Poor handwriting on medical record can cause the medication errors	1	2	3	4
11	Due to weakness of current medical record system, there could be making mistakes like prescribing wrong drug, making wrong diagnosis.	1	2	3	4
**Difficulties in sharing patient records**					
12	I feel that information flow is slow in paper based medical record system.	1	2	3	4
13	Duplication of investigation test can be occurred due to poor record system.	1	2	3	4

**Engagement readiness for EMRs adoption**					
	**Statements**	**Strongly disagree**	**Disagree**	**Agree**	**Strongly agree**
**Potential negative impacts**					
1	The cost for application of EMR is very high and it is not advisable to change current system of manual recording.	4	3	2	1
2	I am lacking ICT knowledge and I prefer current paper based medical record system then EMR.	4	3	2	1

3	To implement a fully computerized EMR system, it would take a long time but nothing of great benefit.	4	3	2	1
4	I worry about the current seamless workflow after changing a new EMR system.	4	3	2	1
**Recognition of benefits**					
5	Application of EMR would help me improve efficiency of medical care.	1	2	3	4
6	EMR system is more secure the privacy of patient information.	1	2	3	4
7	The application of EMR can reduce the waiting time of the patient for medical care.	1	2	3	4
8	The application of EMR have better provision and sharing of timely information between health professionals and hospitals.	1	2	3	4
9	Changing current paper based system to EMR is merely changing of writing with ball-pen to writing with keyboard. No benefit.	4	3	2	1
**Willingness to accept EMR**					
10	I have desire to learn about EMR that I have not understood well.	1	2	3	4
11	I am lacking computer knowledge and afraid to change paper based system to EMR system.	4	3	2	1
12	I would like to participate in the process of development of EMR if I have a chance.	1	2	3	4
